# Physiological, Biochemical and Reproductive Studies on *Valeriana wallichii*, a Critically Endangered Medicinal Plant of the Himalayan Region Grown under In-Situ and Ex-Situ Conditions

**DOI:** 10.3390/plants9020131

**Published:** 2020-01-21

**Authors:** Mohd Asgher, Susheel Verma, Nafees A. Khan, Dhiraj Vyas, Priyanka Kumari, Shaista Rashid, Sajid Khan, Shaista Qadir, Mohammad Ajmal Ali, Parvaiz Ahmad

**Affiliations:** 1 Plant Physiology and Biochemistry Laboratory, Department of Botany, School of Biosciences and Biotechnology, Baba Ghulam Shah Badshah University, Rajouri, Jammu and Kashmir 185234, India; shaistachouhan707@gmail.com; 2 Conservation and Molecular Biology Laboratory, Department of Botany, School of Biosciences and Biotechnology, Baba Ghulam Shah Badshah University, Rajouri, Jammu and Kashmir 185234, India; eremurus@rediffmail.com (S.V.); pk838511@gmail.com (P.K.); sajidkhan717@gmmail.com (S.K.); 3 Plant Physiology and Biochemistry Laboratory, Department of Botany, Aligarh Muslim University, Aligarh 202002, India; naf9.amu@gmail.com; 4 Biodiversity and Applied Botany Division, Indian Institute of Integrative Medicine (CSIR), Canal Road, Jammu, Jammu and Kashmir 180001, India; dvyas@iii.ac.in; 5 Department of Botany, Womens College, Maulana Azad Road, Srinagar, Jammu and Kashmir 190001, India; sqadirs@yahoo.co.uk; 6 Department of Botany and Microbiology, College of Sciences, King Saud University, Riyadh 11451, Saudi Arabia; alimohammad@ksu.edu.sa; 7 Department of Botany, S.P. College, Srinagar, Jammu and Kashmir 190001, India

**Keywords:** ex-situ, fruit set, in-situ, photosynthesis, antioxidants, proline, *Valeriana wallichii*

## Abstract

*Valeriana wallichii*, a perennial herb belonging to family Valerianaceae, is an important medicinal herb of the Himalayan region. The incessant exploitation of nature for meeting the demands of the pharmaceutical industry has put unbearable pressure on its natural habitats. A study on its physiological, biochemical, growth and reproductive attributes was planned. Physiological study revealed that ex-situ (outside their natural habitat) populations faced severe stress as compared to in-situ (natural habitat) plants. The difference in the performance of these habitat plants was related to superoxide and H_2_O_2_ in the leaves. Photosynthetic attributes were increased in in-situ populations. Proline content and its biosynthetic enzymes ornithine aminotransferase, and pyrroline-5-carboxylate reductase showed an increase in ex-situ plants; proline oxidase decreased. Glucose-6-phosphate dehydrogenase, shikimic acid dehydrogenese, phenylalanine lyase, and flavonoids content showed an increment in ex-situ plants. Antioxidants enzyme superoxide dismutase, catalase, ascorbate peroxidase and reduced glutathione showed an increment in ex-situ conditions. Growth and reproductive attributes were more in ex-situ plants. The observations made are suggestive that a comprehensive conservation programme involving in-situ as well as ex-situ strategies will be effective for the conservation and long term survival of the species.

## 1. Introduction

*Valeriana wallichii* DC., a member of family Valerianaceae, is an important medicinal plant of the Himalayan regions [1]. The roots and rhizomes of the *Valeriana* species contain valerenic acids, valepotriates, baldrinal and homobaldrinal, alkaloids and metabolites such as phenolic acids and flavonoids [2]. The plants are medicinally used as diuretic, carminative antispasmodic, anti-constipation, anti-scorpion poison and to improve blood circulation [3]. The biosynthesis of essential oil essentially depends on both the genetic backgrounds and environmental effects [4,5]. It is chiefly found in the temperate Himalaya and cold regions of the northern hemisphere. The habitat of *Valeriana wallichii* is under abiotic stress both due to natural and anthropogenic factors [6]. The species has been a subject of interest due to its application in medicine, variability in morphology, reproductive behaviour and antioxidant properties [7,8].

The study site faces anthropogenic activity that alters the plant habitat, including damage due to developmental activities, particularly, road widening and landslides in the Himalayan region, including Jammu and Kashmir. Moreover, grazing and collection of the material for medicinal use are major anthropogenic threats to the species in nature. The species was also investigated for its reproductive behaviour, as the study of reproductive biology provides information on reproductive bottlenecks [9]. The study may provide information that will help in designing strategies for the conservation of the species in in-situ and ex-situ conditions. Because of the medicinal importance of *Valeriana wallichii*, a study was planned to compare different physiological, biochemical and reproductive parameters both in-situ and ex-situ. It is known that the ex-situ habitats of a species are protected and are away from the natural habitats. Therefore, a lot of significance is associated with the study in comparing the behaviour of the species in both sets of conditions. The data generation on *Valeriana wallichii* is expected to provide important information in recommending the strategy for the conservation of *Valeriana wallichii* to meet the commercial demand.

Initially, plants show several symptoms associated with abiotic stress toxicity, such as decreased seed germination, nutrients content and water uptake together with inhibition of enzymatic activity, and reduced energy supply by inhibiting photosynthesis and energy-releasing catabolic processes. These adverse responses cumulatively result in a reduction of growth and yield in plants [10,11,12]. Chlorophyll fluorescence provides the information related to photosynthetic performance [13,14] and status of other physiological attributes of the plants [15]. The sensitivity of PSII to stress has made this a key technique for knowing the mechanisms of photosynthesis to a changing environment [16]. Chl fluorescence shows alteration under abiotic stress conditions [17]. Recently, it has been shown that the reduction in photosynthesis due to different environmental stresses leads to inhibition of quantum yield of electron transport and electron transport rates that ultimately reduced growth in *Brassica juncea*, *Triticum aestivum* and *Valeriana officinalis* [18,19]. Plants adopt various mechanisms to tolerate stress and these mechanisms include the induction of antioxidant, proline and phenolics/flavonoids metabolisms which act as important scavengers of reactive oxygen species (ROS) [20,21].

Under stressful conditions, the metabolic system of plants responds both in-situ and ex-situ populations. Phenolics, especially flavonoids, are well known H_2_O_2_ scavengers and have an indispensable role in the phenolic cycle which is the main constituent of ROS homeostasis mechanisms [21,22,23]. Rezazadeh, et al. [24] showed that stress tolerance increased with increasing total phenolic and flavonoid compounds in antichoke leaves. The enzymes of the phenylproponaid metabolism were reportedly increased under stress conditions [25].

Proline potentially detoxifies excess ROS, maintains the cellular osmotic environment and protects biological membranes under stress conditions [14,26]. The enzymes of proline biosynthetic pathways have been reported to be upregulated (glutamyl kinase, ornithine aminotransferase (OAT), pyrroline-5-carboxylate reductase (P5C)) or downregulated (proline oxidase) under stressful conditions in *Brassica juncea* and *Withania somnifera* [25,27], depending on their role in proline accumulation. Thus, proline metabolism may be considered as a key tool for protecting photosynthesis and increasing tolerance against stress conditions. Glutathione is known in signaling pathways that facilitate the acclimation of chloroplast processes to highlight and abiotic stress in order to increase yield and stress tolerance under changing environments [27,28,29,30]. Studies have shown that the detoxifying compounds such as reduced glutathione (GSH), proline and phenolics provide tolerance under changing environments [14,21,30,31]. The present study focuses on enhancing our understanding of physiological and biochemical responses of *Valeriana wallichii* under changing climatic conditions, and to assess the approaches to modulate responses for tolerance that are grown under natural habitat (in-situ) and ex-situ conditions. The present work reports the physiological significance of proline and phenylpropanoid/phenolics metabolisms, and GSH in adaptive responses. This work provides the key pathways to target for modulation and enhancing tolerance to protect the photosynthesis and growth of *Valeriana wallichii* under adverse conditions. It establishes the correlation of the reproductive output and the performance of the species in terms of the fruit/seed set and seed germination of the species.

## 2. Materials and Methods

### 2.1. Plant Materials and Growth Conditions

Two populations of *Valeriana wallichii* were studied: one naturally grown in the area of Budhal of district Rajouri, Jammu and Kashmir (J & K), India, at an altitude of 1781 m (amsl) in-situ (natural habitat), and the other growing in ex-situ conditions in Lead Botanical Garden of the Department of Botany, Baba Ghulam Shah Badshah University (BGSBU) Rajouri, India. In-situ plants were collected from an area with altitude of 1781 m (amsl) that receives average annual rainfall of 932 mm and an average annual temperature of 15.8 °C (space coordinates 33°22′ N 74°38′ E), whereas ex-situ plants were grown at an altitude of 1150 m (amsl) that receives an average annual rainfall of 1011.3 mm and an average annual temperature of 21.7 °C (space coordinates 33°23′ N 74°20′ E), respectively. The ex-situ plants were grown in the experimental beds of the Lead Botanical Garden of the Department and not in controlled conditions such as plant growth chamber or glass house. Plant material was collected and immediately stored in liquid nitrogen until further analysis for the in-situ study. For ex-situ conditions, the in-situ grown plants were transferred to the experimental beds and maintained in the Lead Botanical Garden. For the in-situ study, gas exchange and chlorophyll fluorescence were studied in the in-situ location during the bright, sunny 09:00–11:00 a.m. and photosynthetic photon flux density (PPFD) of 1750 µmol photons m^−2^ s^−1^ by applying red/blue as internal light source using an infrared gas analyzer (IRGA, LiCor 6400 XT Lincoln, NE, USA). The in-situ study was carried out in the summer season of 2015 and the ex-situ study in the summer season of 2018. The plant samples were collected from the natural habitat for studying the physiological, biochemical and growth characteristics. The number of replicates used in the study for each treatment was five (n = 5) for the in-situ and ex-situ studies, respectively. The study of plants under ex-situ conditions included transferring the in situ grown plants to the experimental beds and maintaining them for three years at Lead Botanical Garden of the Department of Botany, BGSBU Rajouri, India. After three years of the transplantation to Lead Botanical Garden, a study was planned to compare the performance of the species, both in-situ and ex-situ plants. The treatments were arranged in a complete randomized block design for the in-situ study. The study of photosynthetic characteristics, proline metabolism, phenylpropanoid/phenolics metabolism, oxidative stress and growth attributes were done in the in-situ and ex-situ grown plants respectively. The study of reproductive characteristics (fruit set, seed germination) and other features such as plant dry and fresh weight, leaf area, length of rhizome and length of petiole were studied. The plants grown were maintained under natural day/night conditions with photosynthetically active radiation (PAR) 680 µmol m^−2^ s^−1^ and an average day/night temperature of 21/17 ± 3 °C and relative humidity of 62–70%.

### 2.2. Histochemical Detection of Reactive Oxygen Species; O_2_^−^ and H_2_O_2_ Accumulation

The accumulation of O_2_^−^ was assayed by the histochemical staining method using nitroblue tetrazolium (NBT) to stain the leaves, adopting the method described earlier by Asgher, et al. [32] with slight modification.

For localization of H_2_O_2_, leaves were immersed into 1 mg per mL solution of 3,3-diaminobenzidine (DAB) prepared in 10 mM phosphate buffer (pH 7.8) by using the staining method described by Asgher, et al. [32] with slight modification. Photographs were taken with a digital camera.

#### 2.2.1. H_2_O_2_ Content

The content of H_2_O_2_ was measured using the method given by Okuda, et al. [33]. Fresh leaf tissues (500 mg) were ground in ice-cold perchloric acid (200 mM), and the homogenate was centrifuged at 1200× *g* for 10 min and the supernatant was neutralised with KOH (4 M). Potassium perchlorate (insoluble) was removed by centrifugation for 3 min at 500× *g*. The absorbance of the reaction mixture of 1.5 mL final volume was read at 590 nm.

#### 2.2.2. Lipid Peroxidation

Lipid peroxidation in leaves was determined by the method of Dhindsa, et al. [34] as the content of thiobarbituric acid reactive substances (TBARS). Leaf tissues (500 mg) were ground in 0.25% 2-thiobarbituric acid (TBA) and in 10% trichloroacetic acid (TCA). The mixture was heated for 30 min at 95 °C and then cooled in ice bath and then centrifuged for 10 min at 10,000× *g*. The intensity of colour was read at 532 nm.

#### 2.2.3. Determination of Reduced Glutathione Content

Reduced glutathione (GSH) content was measured by adopting the method of Anderson [35] and was determined by an enzymic recycling procedure in which it was sequentially oxidised by 5′5′-dithiobis-2-nitrobenzoic acid (DTNB) and reduced by NADPH in the presence of GR. Absorbance was read at 412 nm.

### 2.3. Anti-Oxidant Enzymes Activity

The leaf samples (500 mg) were ground in Tris–HCl, in presence of 1,4-dithiothreitol (5 mM), magnesium chloride (10 mM), magnesium acetate (5 mM), ethylenediamine tetra-acetic acid (1.0 mM) polyvinyl pyrrolidone (1.5%), phenyl methane sulfonyl fluoride (1.0 mM) and 1 µg/mL aproptinin. The homogenate was subjected to centrifugation at 10,000 rpm for 10 min and the supernatant collected served as an enzyme source. The extraction buffer for APX was supplemented with ascorbate (2 mM).

Ascorbate peroxidase activity (APX; EC, 1.11.1.11) was determined by following the method described earlier [27]. The superoxide dismutase (SOD, EC 1.15.1.1) and catalase activities (CAT, EC 1.11.1.6), were estimated spectrophotometrically following the methods of Aebi [36].

### 2.4. Phenolics/Flavonoids Metabolism: Enzyme Activities

Activity of glucose-6-phosphate dehydrogenase (G6PDH; EC 1.1.1.49), shikimate dehydrogenase (SKDH; EC 1.1.1.25), phenylalanine ammonia lyase (PAL; EC 4.3.1.5) was studied in leaves after homogenization in 0.1 M KPB (pH 6.5) consisting of 10% PVP, 0.5 mM DTT, and 5 mM β-mercaptoethanol. The homogenate was centrifuged at 12,000× *g* rpm for 45 min at 4 °C and the supernatant collected served as enzyme source. G6PDH activity was measured during assay mixture containing 50 mM Tris–HCl (pH 7.4), 5 mM MgCl_2_, 0.5 mM glucose-6-phosphate, and 0.5 mM NADP. The reaction was started by the addition of enzyme extract, and the increase in absorbance was measured at 340 nm. The SKDH activity was assayed in 0.1 M Tris–HCl buffer (pH 9) consisting of 2 mM shikimic acid, 0.5 mM NADP, and enzyme extract. Increase in absorbance was recorded for over 3 min at 340 nm [37]. The activities of G6PDH and SKDH enzymes were calculated using the molar absorption coefficient of 6.22 mM^−1^ cm^−1^ [37]. PAL was estimated by using the method of Ali, et al. [38].

#### 2.4.1. Estimation of Total Phenolic Content

The total phenolics content of leaf tissues was estimated using the Folin–Ciocalteu reagent using gallic acid as standard, as adopted by Khatun, et al. [39]. Homogenization of the tissues was done in 90% ethanol and centrifuged for 15 min at 10,000× *g*. Aliquot (0.5 mL) was added to diluted Folin–Ciocalteu reagent. Further, 1.25 mL of 7% Na_2_CO_3_ solution was added after incubation. Absorbance was measured at 760 nm after incubation for 90 min at 23 °C.

#### 2.4.2. Estimation of Total Flavonoids

Flavonoid content in the leaf tissues was determined using quercetin as standard [40]. Grinding of the tissue was done in methanol and centrifuged at 12,000× *g* for 20 min. Extract of 1 mL leaf was added to 2,4-dinitrophenylhydrazine (DNPH) (2 mL) and incubated at 50 °C for 50 min. After cooling, 5 mL of potassium hydroxide (1%) in methanol (70%) was added to the reaction mixture. The absorbance was read at 495 nm on spectrophotometer.

### 2.5. Proline Metabolism-Related Enzymes

The leaf samples were extracted using extraction buffer consisting of 0.1 M KPB (pH 7.4), 1 mM pyridoxal-5phosphate, 1 mM EDTA, 10 mM β-mercaptoethanol, and 1% PVPP. The obtained supernatant was used for the enzyme assays. The assay mixture of ornithine aminotransferase (OAT) consisted of 0.1 M KPB (pH 8.0), 50 mM ornithine, 20 mM α ketoglutarate, 1 mM pyridoxal phosphate, and the enzyme extract. After incubating the mixture for 30 min at 37 °C, 10% TCA was added to terminate the reaction. After adding 0.5% o-aminobenzaldehyde, the absorbance of the developed colour was measured at 440 nm.

The pyrroline-5carboxylate reductase (P5C) contained assay mixture of 0.1 M KPB (pH 7.4), 1 mM pyrroline-5-carobxylate, 0.12 mM NADH, and the enzyme extract. The decrease in absorbance was measured immediately after addition of pyrroline-5-carobxylate at 340 nm.

The PROX activity was estimated by adopting the method of Huang and Cavalieri [41]. The increase in absorbance was recorded at 600 nm at 25 °C using proline to initiate the reaction. PROX activity was expressed in U mg^−1^ protein. One unit (U) of enzyme activity was defined as mM DCPIP-reduced min^−1^ mg^−1^ protein.

Proline content was determined spectrophotometrically by adopting the ninhydrin method of Bates, et al. [42] and Asgher, et al. [27]. Fresh leaf samples (300 mg) were homogenized in 3 mL of 3% sulphosalicylic acid. The homogenate filtrate was reacted with acid ninhydrin and glacial acetic acid 1 mL each for 1 h in a test tube placed in a water bath at 100 °C. The mixture was extracted with toluene and absorbance was measured at 520 nm using L-proline as standard.

### 2.6. Measurement of Plant Fresh Weight and Plant Dry Weight

Plants were washed with water after removing from soil. Fresh weight was determined using electronic balance. Plants dry weight was noted after drying the sample in a hot air oven till constant weight.

### 2.7. Measurement of Leaf Area, Length of Rhizome and Length of Petiole

Leaf area was measured by leaf area meter (Biovis leaf Av); rhizome and petiole length was measured manually.

### 2.8. Gas Exchange Parameters

Gas exchanges parameters were studied using IRGA LI-COR 6400XT. Net photosynthetic rate, stomatal conductance, and intercellular CO_2_ were measured by the data analysis programs of the instrument.

### 2.9. Chlorophyll a Fluorescence Measurements

Chlorophyll fluorescence was measured on fully expanded leaves using a portable photosynthesis system (LI-6400, LI-COR, Lincoln, NE, USA) equipped with a fluorescence chamber (LI-6400-40) with modifications as in Bhat et al. [29]. ΦPSII was determined as Fm′–Fs/Fm′, maximal efficiency of PSII by using Fv/Fm and intrinsic efficiency of PS II was by using Fv/Fm as suggested by Maxwell and Johnson [43].

### 2.10. Total Chlorophyll and Carotenoid Content

Estimation of total chlorophyll and carotenoid content was done by adopting the method of Arnon [44]. Dimethyl sulphoxide was used for the extraction from the leaf tissues, and absorbance was taken on a spectrophotometer.

### 2.11. Reproductive Output

For calculating reproductive output, percent fruit set per plant was calculated. This was studied for open pollination in nature and from plants grown in the nursery under ex-situ conditions in hermaphrodite plants. To determine percent fruit set, first, the average number of flowers per plant and then the average number of fruits formed per plant was determined. Percent fruit set was calculated by using the following formula:

(1) $$\%\mathrm{fruit}\;\mathrm{set}=\frac{\mathrm{Average}\;\mathrm{no}.\;\mathrm{of}\;\mathrm{fruits}\;\mathrm{formed}\;\mathrm{per}\;\mathrm{plant}}{\mathrm{Average}\;\mathrm{no}.\;\mathrm{of}\;\mathrm{flowers}\;\mathrm{produced}\;\mathrm{per}\;\mathrm{plant}}\times 100$$

### 2.12. Seed Germination and Seed to Seed Cycle

Seed germination experiments were carried under lab conditions during different months of the year from March to August. The medium used for germinating the seeds was the mixture of sand, garden soil and manure in a 1:1:1 ratio. Experiment was conducted by taking seeds of two sites of in-situ and ex-situ conditions from the hermaphrodite parent plant. Saplings were then shifted in polybags/small pots and after two months transferred to experimental beds for better growth.

### 2.13. Ex-Situ Conservation

In view of the status and magnitude of threat that the species is facing in nature, a number of measures were tried for its conservation under ex-situ conditions. These include raising plants ex- situ from seeds and seedlings collected from two populations of the species from J & K. The seedlings collected and deposited in Lead Botanical Garden of the University were at 5–6 leaf stage. Seeds were germinated in experimental pots and seedlings thus raised then transferred to beds in Lead Botanical Garden.

### 2.14. Statistical Analysis

Statistical analysis was done using GraphPad InStat 3.0 (San Diego, CA, USA). Data are presented as treatments mean ± SE (*n* = 5). SE = standard error; n = number of samples. The mean difference of in-situ from ex-situ was considered statistically significant at *p* < 0.05 (using Student’s *t*-test).

## 3. Results

### 3.1. Superoxide Ion Accumulation in In-Situ and Ex-Situ Grown Plants of Valeriana wallichii

**Figure 1:**
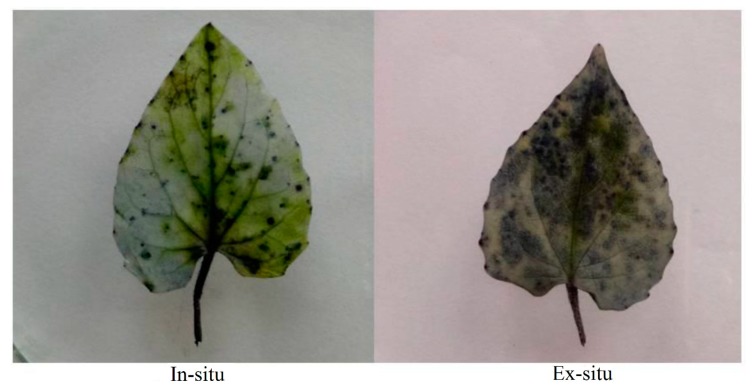
Superoxide ions in in-situ and ex-situ grown plants of *Valeriana wallichii*.

An increased amount of superoxide ion was seen as scattered dark blue spots in the leaves of ex-situ as compared to in-situ grown plants (Figure 1).

### 3.2. H_2_O_2_ Accumulation in In-Situ and Ex-Situ Grown Plants of Valeriana wallichii

**Figure 2:**
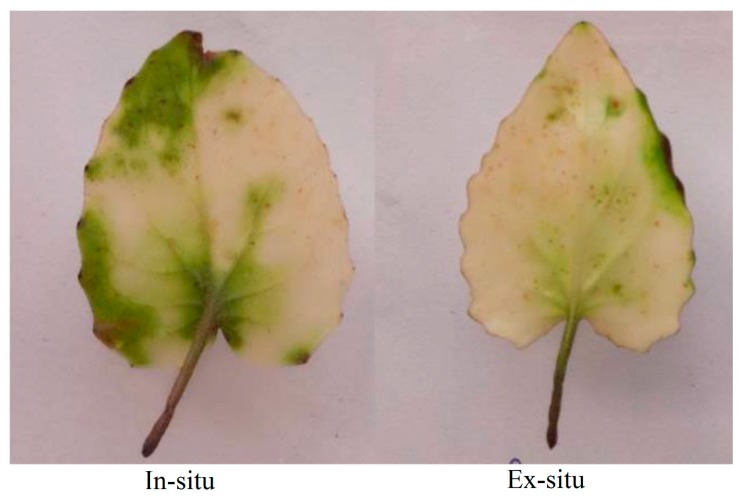
Accumulation of H_2_O_2_ in in-situ and ex-situ grown plants of *Valeriana wallichii*.

An increased amount of H_2_O_2_ was observed as scattered brown spots in the leaves of the ex-situ than in-situ grown plants (Figure 2).

### 3.3. H_2_O_2_ and TBARS Content in In-Situ and Ex-Situ Grown Plants of Valeriana wallichii

Content of H_2_O_2_ and TBARS increased significantly by 1.46-fold and 3-fold in ex-situ and in-situ grown plants of *Valeriana wallichii* (Table 1).

### 3.4. Proline Metabolism Enzymes in In-Situ and Ex-Situ Grown Plants of Valeriana wallichii

The ornithine aminotransferase and pyrroline-5-carboxylate reductase showed an increase of 1.5-fold and 1.1-fold and a decrease of 1.6-fold in proline oxidase in ex-situ grown plants as compared to in-situ grown plants. Proline content significantly increased in ex-situ grown plants by 3-fold than in in-situ plants (Table 1).

### 3.5. Phenolics Metabolismin In-Situ and Ex-Situ Grown Plants of Valeriana wallichii

Glucose-6-phosphate dehydrogenase, shikimic acid dehydrogenese, phenylalanine lyase showed an increase of 2.54-fold, 1.4-fold and 1.3-fold in ex-situ grown plants in comparison to in-situ grown plants. Flavonoid content enhanced by 1.1-fold and total phenolics by 2.4-fold in ex-situ than in in-situ grown plants of *Valeriana wallichii* (Table 1).

### 3.6. Gas Exchange Attributes in In-Situ and Ex-Situ Grown Plants of Valeriana wallichii

**Figure 3:**
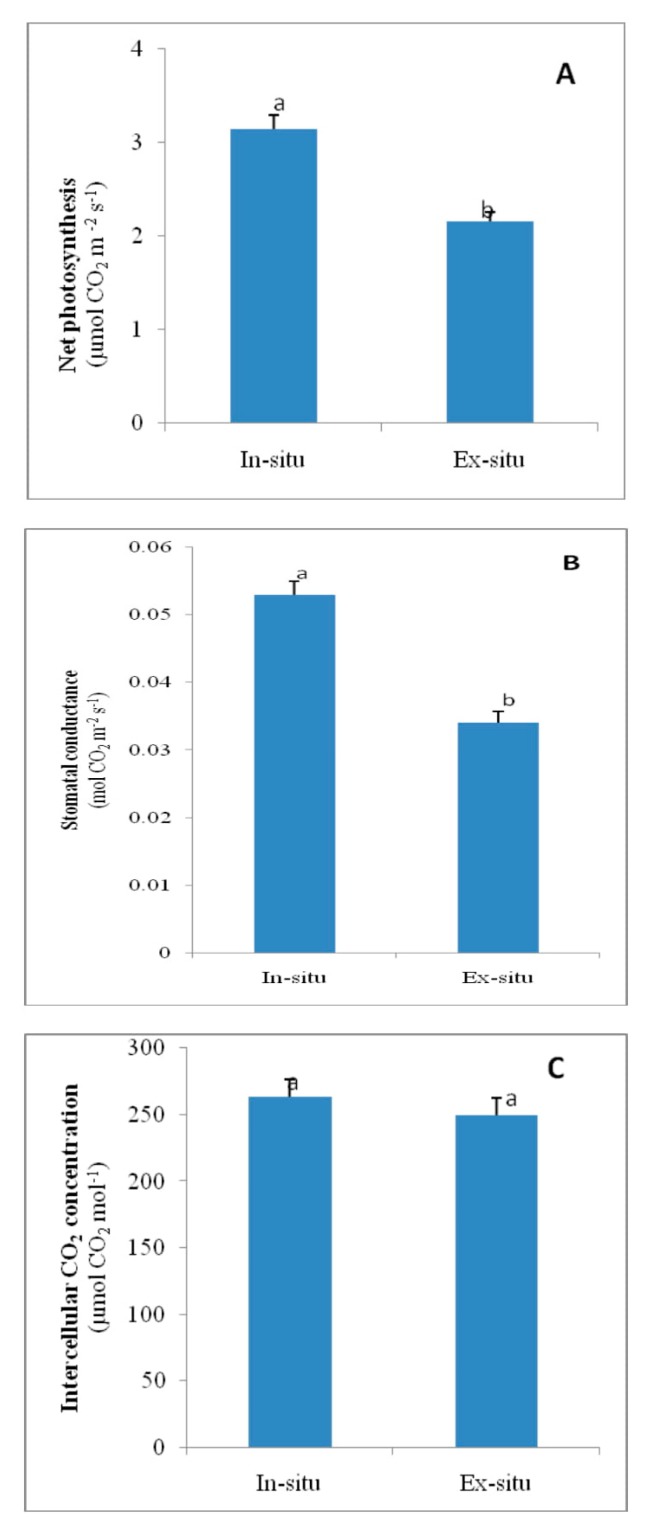
Photosynthesis (**A**), stomatal conductance (**B**), and intercellular CO_2_ concentration (**C**) of in-situ and ex-situ grown plants of *Valeriana wallichii*. Data are presented as treatments mean ± SE (*n* = 5). SE = standard error; n = number of samples. The mean difference of in-situ from ex-situ was considered statistically significant at *p* < 0.05 (using Student’s *t*-test). Data followed by the same letter are not significantly different.

The gas exchange parameters, net photosynthesis, stomatal conductance and intercellular CO_2_ concentration increased by 31.4%, 35.8% and 5.2%, respectively, in in-situ grown plants of *Valeriana wallichii* than in ex-situ grown plants (Figure 3).

### 3.7. Quantum Yield, Chlorophyll Fluorescence and Reaction Centre ETR in In-Situ and Ex-Situ Grown Plants

**Figure 4:**
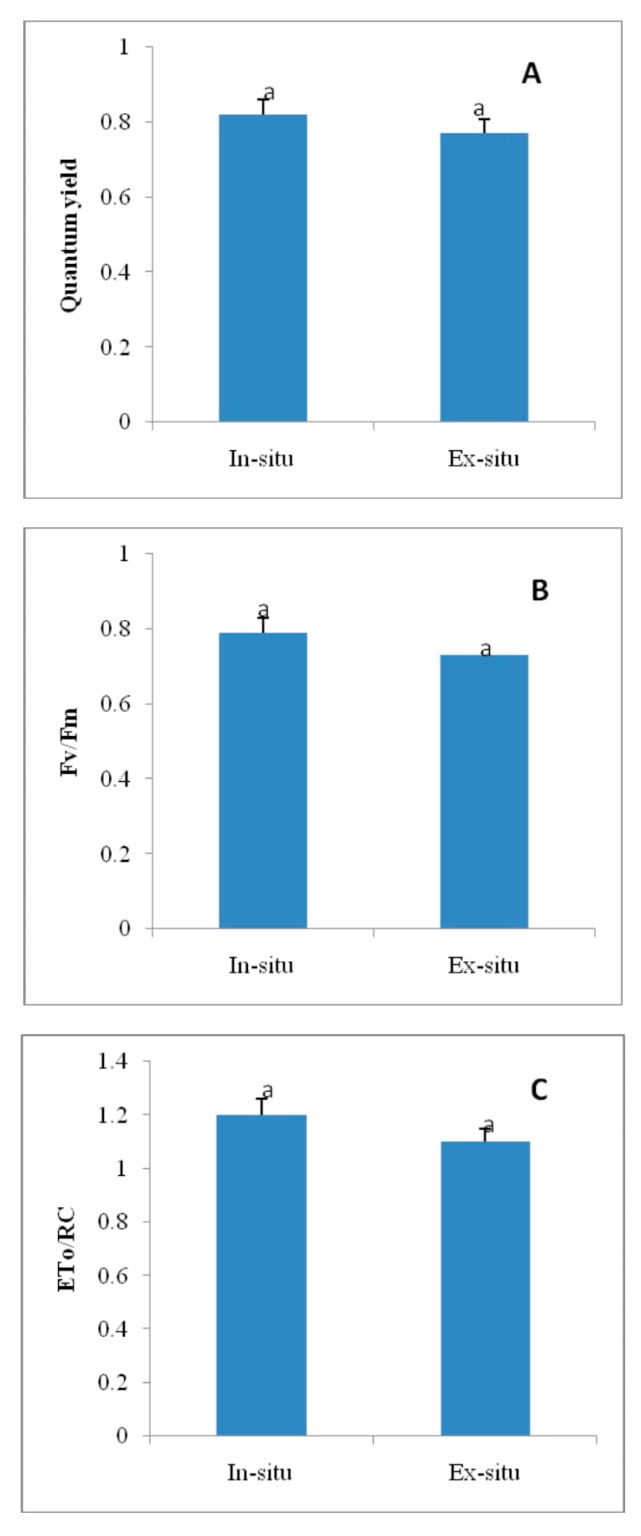
Quantum yield (Φ_PSII_) (**A**), chlorophyll fluorescence Fv/Fm (**B**), and reaction centre ETR (ETo/RC; electron transport flux per active reaction center) **(C)** in in-situ and ex-situ grown plants of *Valeriana wallichii.* Data are presented as treatments mean ± SE (*n* = 5). SE = standard error; n = number of samples. The mean difference of in-situ from ex-situ was considered statistically significant at *p* < 0.05 (using Student’s *t*-test). Data followed by the same letter are not significantly different.

Fluorescence parameters, quantum yield (Φ_PSII_), chlorophyll fluorescence (Fv/Fm) and reaction centre ETR (ETo/RC) were increased by 6.7%, 7.5%, and 14.7%, respectively, in in-situ in comparison to ex-situ grown plants of *Valeriana wallichii* (Figure 4).

### 3.8. Fresh Weight and Dry Weight in In-Situ and Ex-Situ Grown Plants of Valeriana wallichii

**Figure 5:**
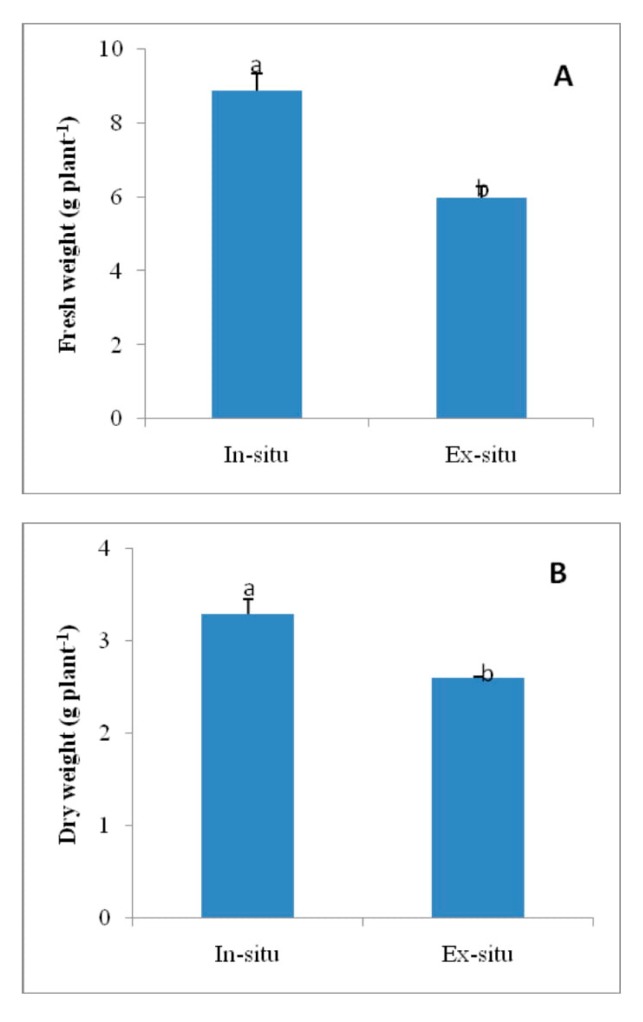
Fresh weight (**A**) and dry weight (**B**) in in-situ and ex-situ grown plants of *Valeriana wallichii*. Data are presented as treatments mean ± SE (*n* = 5). SE = standard error; n = number of samples. The mean difference of in-situ from ex-situ was considered statistically significant at *p* < 0.05 (using Student’s *t*-test). Data followed by the same letter are not significantly different.

Growth attributes, fresh mass and dry mass increased significantly by 32.2% and 20.9%, respectively, in in-situ grown plants of *Valeriana wallichii* than in ex-situ grown plants (Figure 5).

### 3.9. Leaf Area, Length of Rhizome and Length of Petiole in In-Situ and Ex-Situ Grown Plants of Valeriana wallichii

**Figure 6:**
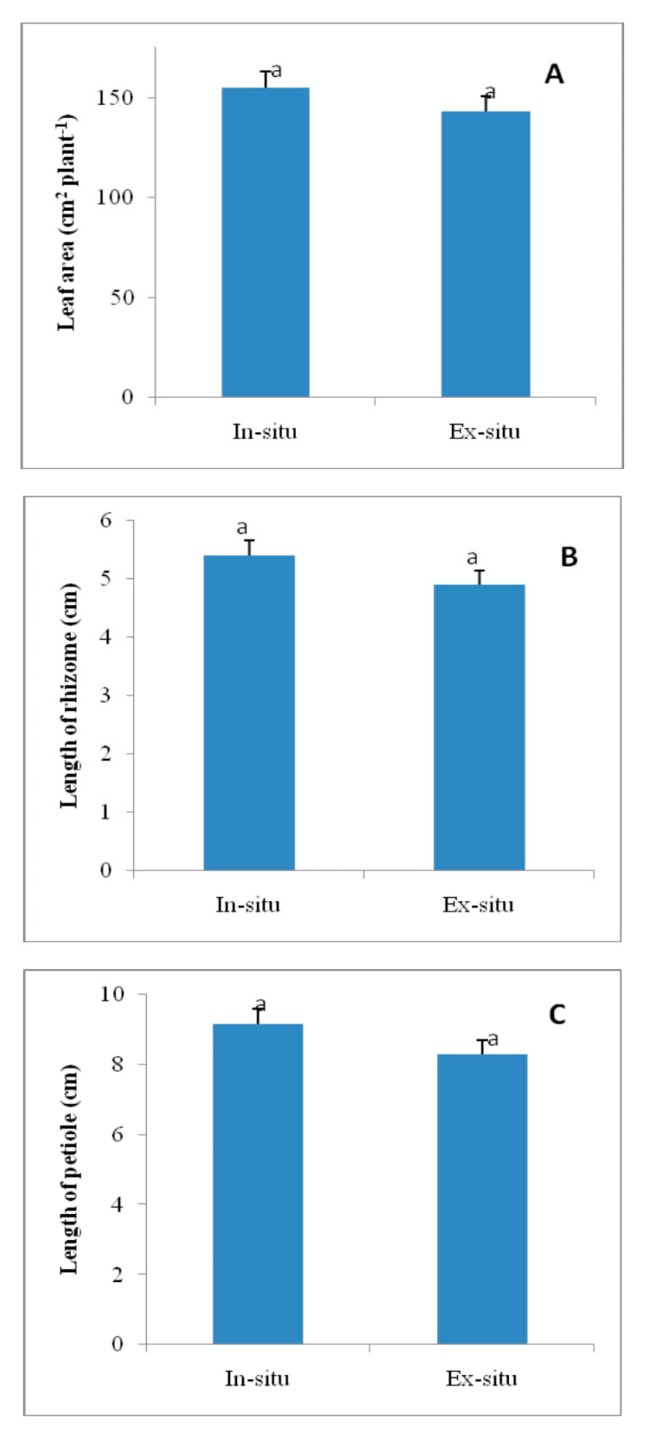
Area (**A**), length of rhizome (**B**), and length of petiole (**C**) in in-situ and ex-situ grown plants of *Valeriana wallichii*. Data are presented as treatments mean ± SE (*n* = 5). SE = standard error; n = number of samples. The mean difference of in-situ from ex-situ was considered statistically significant at *p* < 0.05 (using Student’s *t*-test). Data followed by the same letter are not significantly different.

Leaf area, length of rhizome and length of petiole increased by 1.08-fold, 1.1-fold and 1.10-fold, respectively, in in-situ grown plants of *Valeriana wallichii* than in ex-situ grown plants (Figure 6).

### 3.10. Chlorophyll and Carotenoid Content in In-Situ and Ex-Situ Grown Plants of Valeriana wallichii

Chlorophyll content was found to have decreased by 0.75-fold and carotenoid content increased by 1.5-fold in ex-situ than in in-situ grown plants of *Valeriana wallichii* (Table 1).

### 3.11. GSH Content in In-Situ and Ex-Situ Grown Plants of Valeriana wallichii

GSH content increased by 1.1-fold in ex-situ in comparison to in-situ grown plants of *Valeriana wallichii* (Table 1).

### 3.12. Antioxidants Enzyme in In-Situ and Ex-Situ Grown Plants of Valeriana wallichii

Activity of SOD, CAT and APX increased by 1.2-, 1.1- and 1.5-fold, respectively, in ex-situ grown plants of *Valeriana wallichii* than *in in-situ* grown plants (Table 1).

### 3.13. Reproductive Output in In-Situ and Ex-Situ Grown Plants of Valeriana wallichii

Plants from natural populations (in-situ) showed an average fruit set of 42%. Under ex-situ conditions, the percentage age fruit in hermaphrodite plants was 64.5%. Percent fruit set under different conditions and in different kinds of plants are listed in Table 2.

### 3.14. Seed Germination and Seed to Seed Cycle in In-Situ and Ex-Situ Grown Plants of Valeriana wallichii

**Figure 7:**
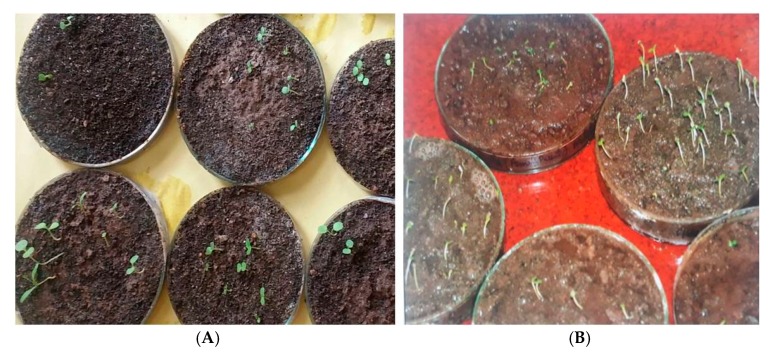
*Valeriana wallichii*: (**A**) In-situ seed germination; (**B**) ex-situ seed germination.

The seeds do not undergo any period of dormancy and germinate soon after falling on a suitable substratum. In nature, it was found that seeds fallen from parent plants start germinating when the plant itself produces new flowers. This was also confirmed by performing seed germination experiments in the lab (Figure 7). Seed germination began in the month of March and continued up to August but no germination occurred thereafter. Conditions were same for both in-situ and ex-situ seed as all the experiments were performed in the lab. Different media or substrata were used for germinating the seeds but the best results were obtained in sand, garden soil and manure mixed in 1:1:1 ratio. Other media tried were farmyard manure and sand in 1:1 ratio and wet filter paper. The germination was epigeal i.e., the radical emerges first and elongation of hypocotyl leads to the elevation of cotyledonary leaves. Seed germination was initiated within 4–8 days after sowing. Seed germination experiments were conducted by taking seeds of hermaphrodite plants. Results of these experiments conducted are summarized in Table 3.

For ex-situ conservation, seeds from as many populations as possible are required to be collected and grown in soil enriched with organic manure to simulate the natural conditions. Seeds can also be preserved in a gene bank at −20 °C. Collection from wild or ex-situ sites should be done in such a manner that the proportion of different sex types is maintained in the populations.

Plants respond fairly well to ex-situ conservation measures. The seedlings that survived were deposited in Lead Botanic Garden for further development. Seed germination was found to be fairly good up to August and completely lost thereafter; better seed germination was achieved between March and August. A total of 1196 seeds were tried for germination between March and August. They showed an overall germinability of 53.2% between March and August. Percentage seed germination in in-situ conditions was found to be 35.6% and in ex-situ was 70.30%. Seeds lost their viability in a period of six months as they showed no germination during the following year. Seedling survival was almost negligible up to June, perhaps due to high atmospheric temperature. Seedlings produced during July and August showed some percentage of survival. The plants thus established successfully entered the reproductive phase during the flowering period. Thus, the seed to seed cycle for the species is one year. In one observation, 100 seeds collected from female plants after open pollination were put to germination, 87 germinated. Out of these, only 17 reached maturity while the rest succumbed. All 17 entered the flowering phase and interestingly turned out to be hermaphrodite.

## 4. Discussion

The present study was designed to investigate the physiological, biochemical, reproductive and growth changes in *Valeriana wallichii*, a threatened medicinal plant of Himalaya based on the in-situ (wild) and ex-situ conditions for obtaining promising stock with the best levels of these attributes. In-situ grown plants showed better photosynthetic and growth response than ex-situ grown plants. This performance of plants is attributed to the more efficient antioxidant metabolism system such as SOD, CAT and APX present in the natural grown plants. The available literature has indicated that the antioxidative metabolism induces an effective defense system against ROS attack [18,32,45]. Moreover, elevated ROS such as H_2_O_2_ resulted in reduced growth and might be due to oxidative damage to the biomolecules. Leaf area, length of rhizome and petiole length were not significantly increased. Ex-situ plants showed more oxidative stress than in-situ plants as these plants were grown in a botanical garden that lacked the in-situ environmental conditions. In *Valleriana wallichii*, the accumulation of superoxide ion and H_2_O_2_ as stress indicators was more in ex-situ than in-situ grown plants, as revealed from the scattered dark blue and brown spots in the present study. These results are in agreement with that of Khan, et al. [46] and Asgher, et al. [32], who observed more accumulation of ROS in mustard plants. The increase in photosynthesis in in-situ grown plants is due to reduction in stomatal and non-stomatal response. It has been shown that water loss via stomata costs photosynthesis by reducing carboxylation capacity and mesophyll conductance. In-situ grown plants were found more effective in increasing photosynthesis and fluorescence parameters, including ETR showing non-significant values. Better photosynthetic performance was mainly due to improved stomatal conductance. Gas exchange parameters such as net photosynthesis and stomatal conductance were increased, which in turn increased growth attributes due to less oxidative stress in these plants when compared to plants in ex-situ conditions. The available literature has shown that the increase in the photosynthetic efficiency of in-situ grown plants was due to an increment in the distribution of N and S to Rubisco in plants [32,47]. Growth parameters, net photosynthesis and stomatal conductance have been observed to be reduced under stress conditions in *Salvia miltiorrhiza* and *Brassica juncea* [32,48]. These findings are in agreement with that of Sehar, et al. [19] where authors have reported a reduction in photosynthesis and growth under stress conditions in *Triticum aestivum*. Reduction in chlorophyll, gas exchange parameters (net photosynthesis and stomatal conductance) and growth characteristics in ex-situ grown plants were due to more oxidative stress in these plants and higher carotenoid synthesis in ex-situ grown plants. Carotenoids are considered as light-harvesting pigments which are involved in protecting chlorophyll and chloroplast. Reports have indicated that phytohormones such as ethylene enhanced photosynthesis by increasing stomatal conductance [46,49].

Secondary metabolites have many fundamental roles in plants and are involved in protecting plants under abiotic stress [20]. Secondary metabolites such as flavonoids with good antioxidant property depend on the structure and substitution patterns of hydroxyl groups. Results have revealed the active participation of carotenoids, proline, phenolics, and flavonoids in ROS management and tolerance mechanisms. The present study confirmed the strong adaptive mechanisms of *Valeriana wallichii* grown in in-situ and ex-situ conditions. Proline is involved in several cellular functions including chlorophyll reconstruction, osmoregulation, stabilization of protein, scavenging of ROS, regulation of NAD/NADH ratio and as a nitrogen source and energy for growth processes [14,26,50,51]. Phenolics also have the ability to remove radical species and also have redox properties [38]. Phenolics have also been shown to be increased on exposure to higher stress in *W. somnifera* [50]. Proline and phenolics metabolism (the metabolic route for the biosynthesis of lignin, flavonoids and phenolics) involvement was comparatively higher which reflected their major role in providing the required osmoticum and antioxidants to the plants under ex-situ conditions. The higher synthesis of total phenolic, flavonoids, and alkaloid contents provides evidence for defense against stress conditions. Other studies published on *Nigella sativa* by Bourgou, et al. [52] and *Mentha pulegium* by Oueslati, et al. [53] showed an increase of phenols under stress saline conditions.

Ornithine aminotransferase, P5C, showed an increase in ex-situ grown plants (except proline oxidase), reflecting their participation in proline accumulation. Similarly, higher proline and activity of enzymes such as OAT and P5C and lower activity of proline oxidase have also been reported earlier [32,47,54]. Both G6PDH and shikimate dehydrogenase (SKDH) are key enzymes for the phenylpropanoid pathway/polyphenolics pathway as they trigger the synthesis of phenolics by providing the precursors. G6PDH has a key role in carbon metabolism and synthesizes the precursor erythrose-4-phosphate for the shikimate pathway, which converts into the aromatic amino acids like phenylalanine which are further converted into the phenolics. Flavonoids content/total phenolics content increased due to smooth functioning of enzymes of phenolics metabolism such as glucose-6-phosphate dehydrogenase, shikimic acid dehydrogenese and phenylalanine lyase. Similarly, there is an increment seen in these enzymes, such as G6PDH, SKDH and phenylalanine lyase, under stress conditions [25]. Higher proline metabolism, phenolics, GSH and carotenoids provide an adaptive mechanism to ex-situ grown plants of *Valeriana wallichii*. The optimal GSH/phenolics/proline accumulation provide tolerance to plants that thrive under natural conditions by acting as osmoprotectants, potent antioxidants that are involved in ROS scavenging. Higher GSH/phenolics/proline accumulation scavenged ROS induced by oxidative stress in ex-situ grown plants. The enzyme of the phenolic biosynthetic pathway triggers the synthesis of phenolics in plants. These secondary carbon metabolites are the end products of primary carbon metabolism. Reports also show that phenolics work in connection with key antioxidants to scavenge ROS [55]. Glutathione is an important metabolite involved in defense and redox homeostasis [56]. It can also act as a donor of electron for ROS scavenging in plant metabolism [57]. Both GSH and proline efficiently provided tolerance to plants against stress [58]. The variations in physiological studies and other studies such as those on the phenolics and proline metabolism of in-situ and ex-situ grown plants might be due to two reasons: first, change in habitat; second, due to chemical phenotypes. In some plants, the chemical phenotypes of various herbal medicines depend on the environment in which they grow [59,60]. Proline protects plants against the metal-induced stress by increasing the activity of antioxidants and an improved level of reduced GSH provides a reducing environment in the cell. Higher contents of proline, phenolics and GSH are adaptive responses of these plants, showing that these plants encountered severe stress [61,62,63,64].

**Figure 8:**
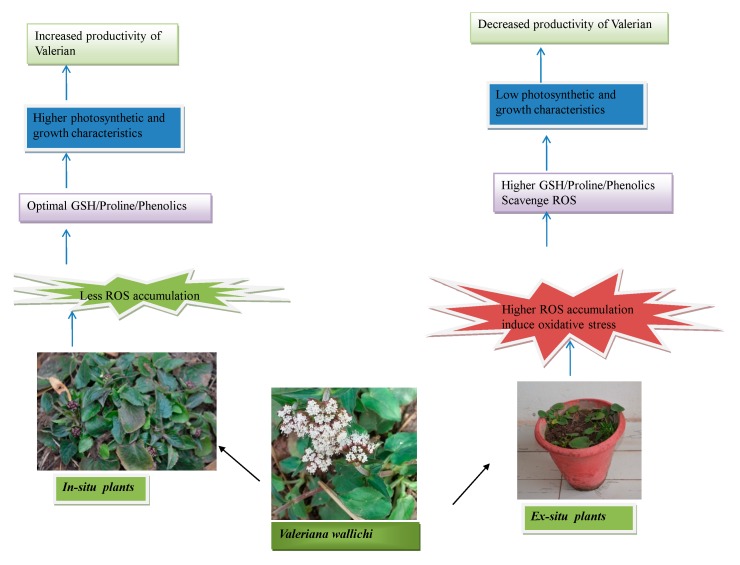
Mechanism of plant metabolism in in-situ and ex-situ grown *Valeriana wallichii*.

In-situ grown plants showed less accumulation of ROS, resulting in optimal metabolite accumulation. Consequently, such plants registered higher photosynthesis and growth characteristics which might be a better option for obtaining the desired medicinal compound. On the other hand, ex-situ grown plants showed higher ROS accumulation, indicating oxidative stress and higher metabolitie accumulation (Figure 8).

As far as the reproductive biology of *V. Wallichii* is concerned, it was found that the reproductive output of plants grown under ex-situ conditions was more than in-situ grown plants, pointing towards a pollen/pollinator limitation in in-situ populations of the species. It was also revealed from seed germination experiments that seeds raised in ex-situ conditions showed better germination ability than in-situ raised seeds, which also resulted in an increased reproductive output of the species. Ex-situ conservation is complementary to in-situ methods as it provides protection against the extinction of threatened plant species and execution in recovery programmes of endangered species. Although the reproduction performance of the species appeared to be better in ex situ conditions, in-situ conditions provided appropriate conditions for evolution which were exhibited by the physiological, biochemical and growth changes in such plants. The study confirmed that in situ and ex-situ practices provide a reservoir to conserve plants, and the ex-situ mode has saved the species from extinction. Further, better reproductive performance in ex-situ conditions was attributed to better pollination as the species is insect-pollinated and insects are abundant in ex-situ conditions [7].

## 5. Conclusions

It may be concluded that ex-situ grown plants face severe oxidative stress due to the accumulation of ROS that might interfere with the photosynthesis and growth response. Higher proline, phenylproponaid metabolism, GSH and carotenoid provided an adaptive mechanism to ex-situ grown plants of *Valeriana wallichii.* It may be said that in targeting these biosynthetic pathways, the tolerance property of the plants against abiotic/unfavourable conditions can be improved. Reproductive performance under ex-situ conditions appeared better than in-situ, which was due to better insect pollination under ex-situ conditions, as pollinator availability was better in ex-situ than in in-situ conditions.

## Figures and Tables

**Table 1:** Content of H_2_O_2_, TBARS and GSH, activity of catalase (CAT), superoxide dismutase (SOD), ascorbate peroxide (APX), total phenolics content, proline content, ornithine aminotransferase, pyrroline-5-corboxylate reductase, proline oxidase, glucose-6-phosphate dehydrogenase, shikimic acid dehydrogenese, phenylalanine lyase, flavonoids content, carotenoid content and total chlorophyll content in in-situ and ex-situ grown plants of *Valeriana wallichii*.

Parameters Studied	In-Situ Grown Plants	Ex-Situ Grown Plants	*p*-Value
H_2_O_2_ content (nmol g^−1^ FW)	48.2 ± 5.05 b	70.5 ± 6.8 a	<0.05 *
TBARS content (nmol g^−1^ FW)	4.8 ± 0.9 b	14.5 ± 1.2 a	<0.001 ***
GSH content (nmol g^−1^ FW)	280.3 ± 19 a	307.2 ± 21 a	0.3700
Catalase activity (mg protein min^−1^)	107.2 ± 5.36 a	119.6 ± 5.98 a	0.1611
Superoxide dismutase activity (mg protein min^−1^)	4.8 ± 0.24 a	5.3 ± 0.26 a	0.1953
Ascorbate peroxidase activity (mg protein min^−1^)	0.9 ± 0.04 b	0.13 ± 0.06 a	<0.001 ***
Total phenolics content (mg g^−1^ DW)	0.5 ± 0.08 b	1.2 ± 0.07 a	<0.001 ***
Proline content (mg g^−1^ FW)	4.1 ± 0.92 b	12.2 ± 1.2 a	<0.001 ***
Ornithine aminotransferase (U mg protein min^−1^)	6.2 ± 0.99 a	9.3 ± 1.1 a	0.0695
Pyrroline-5corboxylate reductase (P5C) (U mg protein min^−1^)	10.4 ± 1.2 a	12.1 ± 1.3 a	0.3647
Proline oxidase (U mg protein min^−1^)	0.05 ± 0.006 a	0.03 ± 0.007 a	0.0619
Glucose-6-phosphate dehydrogenase (U mg protein min^−1^)	7.2 ± 1.0 b	18.3 ± 2.1 a	<0.01 **
Shikimic acid dehydrogenese (U mg protein min^−1^)	2.2 ± 0.5 a	3.1 ± 0.6 a	0.2585
Phenylalanine lyase (U mg protein min^−1^)	2.6 ± 0.6 a	3.4 ± 0.4 a	0.2995
Flavonoids content (mg g^−1^ DW)	0.40 ± 0.008 a	0.45 ± 0.07 a	0.001
Total chlorophyll content (mg g^−1^ FW)	2.4 ± 0.3 a	1.8 ± 0.2 a	0.1347
Carotenoid content (mg g^−1^ FW)	0.6 ± 0.02 b	0.9 ± 0.007 a	<0.001 ***

Data are presented as treatments mean ± SE (*n* = 5). SE = standard error; n = number of samples. Different letters show significant difference at *p* < 0.05 (using Student’s *t*-test). *, ** and *** present significant, very significant and extremely significant, respectively.

**Table 2:** Reproductive output under in-situ and ex-situ conditions.

S. No.	Kind of Treatment	No. of Flowers Under Observation	No. of Fruits Formed	No. of Plants to Which the Flowers Belonged	Percent Fruit Set
1.	Open pollination in-situ conditions	3647	1533	21	42.03%
2.	Open pollination under ex-situ conditions	17,241	11,125	53	64.5%

**Table 3:** Seed germination in in-situ and ex-situ conditions.

Activity	March		April		May		August	
	In-Situ	Ex-Situ	In-Situ	Ex-Situ	In-Situ	Ex-Situ	In-Situ	Ex-Situ
No. of seeds put for germination	150	150	150	207	150	100	150	139
No. of seeds germinated	95	116	65	157	45	73	9	76
